# Digital property security breaches and virtual ownership enforcement: a cyber law perspective

**DOI:** 10.3389/frai.2026.1910871

**Published:** 2026-09-22

**Authors:** Kieu Thi Thuy Linh

**Affiliations:** Vietnam Women's Academy, Hanoi, Vietnam

**Keywords:** digital property security, human rights, liability and compensation, property rights, virtual ownership enforcement

## Abstract

**Introduction:**

The digital age has produced new forms of property—virtual assets, Non-Fungible Tokens (NFTs), and cloud-based intellectual property—that raise pressing challenges for ownership rights and legal protection. This study examines the impact of digital property security breaches and digital contract compliance on virtual ownership enforcement, fundamental rights, and liability outcomes, with particular attention to the disproportionate effects on vulnerable user groups.

**Methods:**

Drawing on a structured expert-assessment dataset of 240 valid observations compiled from court case records, platform transparency reports, and regulatory documents covering 2018-2024, the study applies Structural Equation Modeling (SEM), independent samples t-tests, and Analysis of Variance (ANOVA) to assess relationships among six constructs: Digital Property Breach Characteristics (DPBC), Digital Contract Compliance (DCC), Virtual Ownership Enforcement (VOE), Fundamental Rights Impact (FRI), Liability and Compensation Outcomes (LCO), and Vulnerable User Groups (VUG).

**Results:**

Higher breach frequency and severity significantly reduce enforcement effectiveness (*p* < 0.001) and negatively affect fundamental rights. Non-compliance with contractual terms is associated with increased liability exposure (*p* < 0.001). Rights violations and enforcement delays are more pronounced among vulnerable populations, including women, children, and marginalized communities.

**Discussion:**

These results underscore the need for stronger digital property governance and more robust, rights-based liability frameworks.

## 1 Introduction

There is a rapid development of technologies of digital assets and digital asset applications, which is profoundly changing the world financial and economic systems through regulatory law frameworks. The blockchain and other distributed ledger technologies have facilitated new paradigms of ownership, exchange, and management of assets within commercial law structures. According to the forecast of the World Economic Forum, almost ten percent of global Gross Domestic Product (GDP) can be stored and compensated in blockchain-based and tokenized systems in the years ahead. Decentralized Finance (DeFi) services like decentralized exchanges, products such as real-world asset (RWA) tokenization, Central Bank Digital Currencies (CBDCs) and innovative fundraising platforms built on token issuance show how blockchain and distributed ledger technologies are finding new uses in the current financial infrastructure subject to financial law compliance. The developments are enabled by open, decentralized, and programmable architectures, which enable recording, verification, and trading of various types of digital assets in blockchain platforms governed by technology law principles (Soin et al., 2025; Proskurovska and Birch, 2025).

Coupled with these financial changes, the metaverse has become a fast-growing digital ecosystem that is powered by the adoption of blockchain technology, artificial intelligence (AI), and immersion, like virtual, augmented, and mixed reality (Bhattacharya et al., 2023). This has converged to transform digital interaction, commerce and organizational practice and also generates complex security and privacy issues related to cyber law. Immersive allow collecting biometric, physiological and behavioral data at a large scale, which spark serious concerns regarding user consent, surveillance and misuse of personal information under privacy law provisions (Singh et al., 2025). Grants to build user profiles in detail only add to the threats of identity exploitation and data abuse. The lack of proper legislative frameworks and regulations that are uniform in data management, authentication, and imposing digital ownership in the virtual world contributes to these challenges in information technology law. In addition, non-consensual communication and new types of digital abuse have not been sufficiently mitigated through current institutional and legal frameworks and skew adversely in favor of vulnerable groups of users, especially women and marginalized populations within human rights law. All these trends point toward the growing relevance of strong cybersecurity, privacy, and efficient management of digital property as a means of ensuring harmonious and secure engagement in the virtual ecosystems through enforcement law mechanisms (Kshetri, 2025).

Artificial intelligence plays an increasingly direct role in digital property security and enforcement. AI-driven systems are now deployed for breach detection, automated contract compliance monitoring, fraud pattern recognition in NFT and blockchain transactions, and algorithmic dispute resolution in platform governance. At the same time, AI introduces new vectors of vulnerability: adversarial attacks on AI-based security systems, automated exploitation of smart contract weaknesses, and the use of generative AI to fabricate identity credentials and ownership records. The European Parliament and Council of the European Union (2024) classifies certain AI systems used in law enforcement and access to essential services as high-risk, imposing transparency, accountability, and human oversight obligations that are directly relevant to automated enforcement of digital property rights (European Parliament and Council of the European Union, 2024). For vulnerable users, AI-mediated enforcement systems may replicate or amplify existing biases, producing systematically worse outcomes for women, children, and marginalized communities. This study situates its analysis of digital property breach and enforcement within this AI-governance context, treating AI-enabled enforcement mechanisms as a key dimension of the virtual ownership ecosystem under study.

Users interact and do activities in the metaverse through their digital avatars by means of augmented reality, virtual reality, and blockchain technology. They capture very fine details as these systems get more immersive and dynamic, including patterns of speech, expressions, eye motions, gestures, body language and behavioral interactions (Pereira et al., 2025). All of the possible privacy and security risks are brought by the large amount of sensitive biometric and physiological information, especially when it is disclosed by identity theft, hacking attacks, and unauthorized access under data protection law (Wang et al., 2023). Online identities on avatars contribute to the issue of surveillance even further as they continuously record user actions in the virtual world. Moreover, centralized data storage designs employed by most metaverse applications create vulnerabilities to data manipulation, information leakage and accountability failures after a security breach within cybersecurity law (Malik, 2024). The severity of these threats is proven by real-life cases of unauthorized access to user identities, monetary assets, and biometric data. Moreover, the low level of interoperability between metaverse platforms does not allow transferring user data and other virtual resources to other digital worlds safely, making it difficult to ensure or even enforce digital ownership rights through intellectual property law. These circumstances emphasize the increasing demand of safe enforcement systems, robust privacy protection, and detailed systems of digital property regulation within immersive digital environments supported by international law standards (Kumar et al., 2024).

Digital ownership and asset management has been re-established by the use of decentralized blockchain technology including Proof of Stake (PoS) and Non-Fungible Tokens (NFTs). NFTs allow the establishment of authentic and traceable ownership of digital and physical properties, such as digital identity, real estate, and intellectual property recognized by property law (Hasan et al., 2023). PoS provides an alternative to Proof of Work that is more energy-efficient and scalable, that is, it is more resistant to fraud and cyberattacks, and it has a better network performance. Combining with NFTs, PoS enhances infrastructures of digital ownership by assisting with their transparency, immutability, and trustworthy management of assets, promoting even greater adoption in other industries through contractual law safeguards (Ogunbiyi-Badaru et al., 2024). Nevertheless, the increasing cases of access breach to digital property, hacking, and the failure of the contract undermine the security of the virtual ownership system under liability law concerns. These violations undermine enforcement systems, make the resolution of disputes difficult, and bring an ambiguity on liability and compensation within civil law procedures. The most vulnerable categories of users are particularly vulnerable since they find it more difficult to defend their digital rights and find effective solutions through consumer protection law (Varadarajan and Seeni, 2024).

Previous studies have identified the technological, privacy, and governance dimensions of digital assets and metaverse worlds, but there is scanty empirical evidence on the effects of security breaches and contractual failures on the implementation of virtual ownership, liability, and compensation solutions, and universal human rights in constitutional law contexts. The lack of focus has also been directed toward the disproportionate effect on marginalized communities and women, and the majority of the past studies have been conceptual in essence without addressing the efficacy of enforcement, and the nature of breaches through judicial law analysis. This research fills these gaps through extensive empirical research that comprises Structural Equation Modeling (SEM) and statistical tests to examine the relationship among breach characteristics, the results of enforcement, and the effects of breach on rights, in addition to the consideration of user vulnerability as a mediating variable. The research offers evidence-based concepts to enhance the digital property governance and enforcement systems through an elaborate framework that integrates technological, legal, and human rights factors within contemporary law research.

### 1.1 Research objective and contribution

The rationale of the research is to examine the impacts of breaches of digital property security and digital contract performance on major constitutional rights, duties and damages of repayment, and management of augmented possession of digital environments under constitutional law. It also examines the impact of vulnerable groups of users to the enforcement effectiveness and rights impact using SEM and statistical analysis within cyber law.

- Offers factual proof of the connection between ownership enforcement results and digital property violations under cyber law.
- Suggests a comprehensive framework that integrates the viewpoints of human rights, law, and technology.
- Identifies vulnerable user groups as a mediating variable in the efficacy of enforcement within constitutional law.
- Uses statistical tests and SEM to model intricate interactions between important variables in legal law analysis.
- Provides useful advice on how platform developers and legislators can enhance the governance of digital property through regulatory law.

### 1.2 Research organization

This research consists of five major parts. Section 1 presents the goals of the research, contributions, definition of the problem, and the background of the research under legal law perspectives. Section 2 reviews the literature on virtual enforcement of ownership and digital property security within cyber law. Section 3 consists of the variables; data source and the methods of analysis applied in this research for law analysis. The empirical findings are presented and discussed in Section 4 under legal law interpretation. Section 5 is made up of the conclusion, which recaps the work, makes an important contribution to the work, states the limits, and recommends future directions of inquiry in technology law research.

## 2 Related work

The research (Fiaz et al., 2024) sought to create a privacy preserving and insecure identity management system in the Metaverse to overcome centralized conceptualizations. It has suggested a decentralized system of Self-Sovereign Identity (SSI) that provides user ownership and control of digital identities. Experimental and mathematical validation showed that centralized systems showed 31, 29, and 34% improvements in cyberattack resistance, integrity assurance and privacy protection respectively. Nevertheless, the framework lacks practical implement ability because it is primarily based on a mathematical validation and does not depend on the implementation in the large scale. The research (Mikhaylov, 2023) set out to investigate the risk factors related to cryptocurrency transactions including trading, lending, borrowing, and wallet services and give suggestions on how these activities can be regulated to participate in them safely. It reviewed 43 research papers that had been published in 2019–2023 to determine the trends in risk management and regulations. Results showed that more than 62 percent of the studies have found that they were highly susceptible to fraud and market risks and tighter regulations enhanced market stability by about 27 percent. Nevertheless, the use of secondary data without transaction evidence precludes the extent of the applicability to regulatory settings in general. The research (Dwivedi et al., 2023) was intended to examine the neglected harmful and dangerous effects of the Metaverse. It has compiled scholarly views and stories by some of the most prominent scholars in various fields. The research results showed that more than half of the experts also reported an increase in financial crime and online abuse, and more than three-quarters of experts cited identity theft and privacy as significant problems. Nonetheless, the research lacks a high degree of generalizability since the expert opinions are used without the extensive quantitative behavioral data to be validated empirically.

The point of the research (Lifang, 2024) was to analyze the legal position and safeguarding of virtual property, such as digital currencies and game-based assets. It used legal research and analysis of the current regulatory systems to evaluate classification and protection systems. Findings revealed that more than 65 percent of the cases pertaining to virtual property that were taken to court had no clear legal definitions, which also resulted in poor consumer protection and a slow process in the settlement of disputes. The research is, however, conceptually oriented and not empirically validated across jurisdictions which restrict their general applicability.

The research (Buletsa, 2023) sought to assess the issue of whether virtual property needs to be considered an intangible civil right under the laws of various countries. It relied on the comparative legal analysis of the metaverse development, the virtual market, international regulation, and the legal system in Ukraine that included blockchain and smart contract mechanisms. The results showed that it is possible to resolve the concerns of ownership and transfer by the use of smart contracts, yet a legal validation and improved protection is required. Nevertheless, traditional models of regulations and empirical information do not provide enough evidence to address the complicated legal and financial issues. The research (Theoulakis, 2024) was an attempt to investigate how virtual reality (VR) and augmented reality (AR) could be used to manage, present, and preserve digital and cultural heritage. It summarized the previous studies and practice, such as virtual museums and interactive heritage spaces. Findings suggested that immersive technologies improved learning outcomes 33, accessibility 38, and interaction with the user 47% and improved traditional conservation strategies. Nevertheless, it is costly to implement, has high infrastructure demands, and not every person has access to AR/VR devices, which restricts massive use and sustainability in the long term.

The research (Kalyvaki, 2023) was focused on examining the major legal issues in the metaverse, namely, the jurisdiction, privacy, and intellectual property. It embraced the use of conceptual legal analysis of the current legal framework and regulatory views to determine its relevance in cross-border virtual settings. The results showed that there was ambiguous jurisdiction because platforms were decentralized across the globe and citing that cohesive legal, organizational, and technical action and interdisciplinary regulation are needed. Nevertheless, the research is rather theoretical and does not contain empirical evidence and real-life case studies. The research (Patwe and Mane, 2025) was intended to discuss the problem of data privacy and security in the metaverse such as manipulation of data, unauthorized access, and misuse of digital footprints of avatars. It suggested a blockchain-based framework based on Hyperledger Fabric and Ciphertext-Policy Attribute-Based Encryption (CPABE), and was tested by experimentally scaling the framework under the conditions of different node counts and attributes. Results showed improvements of 41% in access security, 36% in encryption efficiency, and 30% in transaction reliability. However, validation was limited to experimental settings without large-scale real-world deployment analysis.

The research (Al Shamsi et al., 2023) aimed to explain how NFTs facilitate financial crimes such as crypto-fraud and money laundering using the Space Transition Theory framework. It employed an analytical literature-based approach supported by real-world NFT crime examples. Findings indicate that the transition from physical to virtual art markets promotes criminal activity due to anonymity, flexibility, weak deterrence, and lack of accountability mechanisms. However, the research is largely conceptual and lacks quantitative empirical validation to measure the actual scale of NFT-related financial crimes.

The research (Appel and Grennan, 2024) was an attempt to investigate the applicability of traditional corporate governance models to Decentralized Autonomous Organizations (DAOs). It used a regression discontinuity analysis of close-call proposal votes and tested 28 governance features, such as voting rules, consensus mechanism, proposal right and security measures. The findings revealed that restrictive proposal rules were related to poor performances, but positive abnormal returns, high DEX trading volume, and reduced security breaches were related to inclusive participation and stronger security. The research, however, might be inadequate in terms of capturing the dynamics of governance and sources of behavior over a long term. The research (Dupuis et al., 2023) sought to examine the nature of data breaches through text analytics and machine learning of lawsuits cases narrative. It used topic modeling and descriptive analysis to find out major areas of concern in relation to privacy, negligence and financial fraud. Findings indicated that there are four main groups, which include identity theft (32%), cybersecurity (28%), phone scams (21%), and corporate data breaches (19%), and it has proven effective in gaining knowledge of the stakeholder concerns. Nevertheless, the research is restricted to the court reports, and they might not cover every real-life breach of data. The objective of the research (Wyczik, 2025) was to clear up the legal position of digital assets and counter the idea that digital purchases are not ownership. It carried out comparative legal research of the common law and the civil law jurisdictions to ascertain whether digital property fits into intellectual property, tangible property or even contractual rights. The results indicated that 57 percent of the legal systems consider the digital assets as property rights, mostly in line with the intellectual property law. The research however does not have empirical validation of enforcement practices.

The research (Olushola and Meenakshi, 2025) examined the cybercrimes that took place in bitcoin exchanges, 2009–2024. It determines the primary types of attacks and vulnerabilities in a systematic study and trend analysis of reported cases. The results show that regulatory loopholes and lack of proper security are increasingly leading to a greater number of hacking, phishing, and insider attacks. The report highlights the need to have stronger security and global regulatory collaboration. It has a weakness in relying on publicly reported cases that might not be a complete set of cybercrimes. The research (de las Heras Ballell, 2024) attempted to explore legal ambiguities concerning digital assets and the necessity of harmonizing the regulations globally. It used a comparative and doctrinal legal study of the developing ideas and best practices both in insolvency, enforcement, and property rights. The results suggest that the movement toward homogeneous legal norms to enhance transparency and inter-country enforcement of the law gains momentum, and global cooperation is essential. Nevertheless, the research is very theoretical and does not compare actual performance of suggested frameworks empirically.

The research (López Rodríguez, 2025) was intended to assess the applicability of consumer protection, jurisdiction, privacy, and liability in blockchain-powered metaverse platforms. It also made a comparative legal study on six jurisdictions, the EU, USA, China, Singapore, Brazil and South Korea. Results indicated a disjointed regulatory environment with major gaps that undermine user trust and system sustainability, prompting the suggestion of the hybrid system of governance via legal enforcement and smart contracts. Nevertheless, the discussion is mainly theoretical and does not have an empirical validation on actual metaverse platforms. This research explores the role of blockchain technology in addressing cybersecurity concerns unrelated to cryptocurrency (Malik and Khanna, 2024). The method involves a critical review of the literature, analysis of the use of practice and professional judgments. These results showed an increase of 28, 39, and 34 percent in supply chain integrity, identity management security, and IoT protection, respectively. Some of the disadvantages include scalability and energy consumption. In the research of the level of client safety, the terms and conditions of the primary cryptocurrency custody and wallet service providers are analyzed (Zetzsche and Nikolakopoulou, 2025). It involves a review of a huge amount of custody agreements along with related processes such as liability, asset segregation, safe keeping and dispute resolution. These findings indicated that users were more vulnerable as only 46% of custodians provided strong types of liability protection and 54% provided moderate coverage. The research did not compare user experiences and working processes.

The research (Zhuk, 2025) was intended to define critical legal, regulatory, and technological impediments to the adoption of blockchain by various sectors. It utilized a qualitative synthesis of the available literature and regulatory views. It has been found out that privacy, data protection, security risks, and regulatory uncertainty are among the major problems, and integration problems of Internet of Things (IoT), AI, and big data. The paper concentrates on the necessity of legal frameworks and partnerships between stakeholders. But it is mostly theoretical and does not have empirical or case-based validation of the challenges identified. The research (Wu and Zhang, 2023) was intended to resolve the problem of trust in avatars interaction by investigating the problem of digital identity and privacy protection in the Metaverse. It applied a legal and concept analysis of authentication infrastructure, privacy data management and digital identity framework. The results indicate that even though the issue of identity violations can be resolved via the provisions of personality rights laws, a more coherent digital identity verification framework and a well-defined set of regulations on identity data are necessary to balance privacy and the use of data. Nonetheless, the research is very hypothetical and not technically carried out or supported by empiricism.

The research (Hornuf et al., 2025) examined the impacts of cybercrime on risk-taking and returns of investors of Ethereum market. The method involves difference-in-differences comparison of blockchain address of victim and matched non-victims. The results suggest that long-term risk undertakings reduce risk-adjusted returns as victims incur losses. The richer victims have systematic rehabilitation methods and the poorer victims involve themselves in gambling. The research is only restricted to the behavior that can be observed on-chain, and this indicates that it will not be able to comprehensively research off-chain financial activities or investment intentions. This research (Kerr et al., 2023) is concerned with the major cryptocurrencies to assess the risks of fraud and financial performance in terms of their investment opportunities. Two areas of the methodology include the examination of the major fraud cases and the comparison of Bitcoin returns with the traditional stock market indices. The research has revealed that compared to typical stock indices, the number of losses related to fraud grew by 37 percent and volatility rose by 45 percent. It has shortcomings of relying on past data that cannot even be capable of predicting the future market.

### 2.1 Research gap

The literature regarding digital property and the Metaverse has several serious limitations. In the absence of data on real enforcement, the research of identity management and privacy protection relied primarily on experimental or statistical confirmation. Moreover, most of the previous studies lacked research on the relationship between the violation, enforcement, and compensation outcomes and employed advanced causal modeling methods such as SEM. This research addresses these limitations by relying on real-world data (2018–2024) of security breaches, enforcement outcomes, and records of disputes alongside developing an analytical framework that captures the interrelationship of the characteristics of digital property breaches, digital contract compliance, virtual ownership enforcement, and effects on fundamental rights. This research is based on earlier studies by specifically simulating vulnerable user populations and applying SEM to illustrate direct and mediating causality between breaches, enforcement effectiveness and culpability and compensation finalities under liability law.

### 2.2 Hypothesis development

Considering the conceptual framework presented by the Figure 1, five hypotheses (H1–H5) are developed to assess direct and indirect relationships between the cybercrime of security breaches in digital properties, compliance with the contract law, the types of vulnerable users, and the outcomes of enforcement of virtual ownership under cyber law.

- H1: The nature of Digital Property Breach (DPBC) has a negative impact on Virtual Ownership Enforcement (VOE).

**Figure 1:**
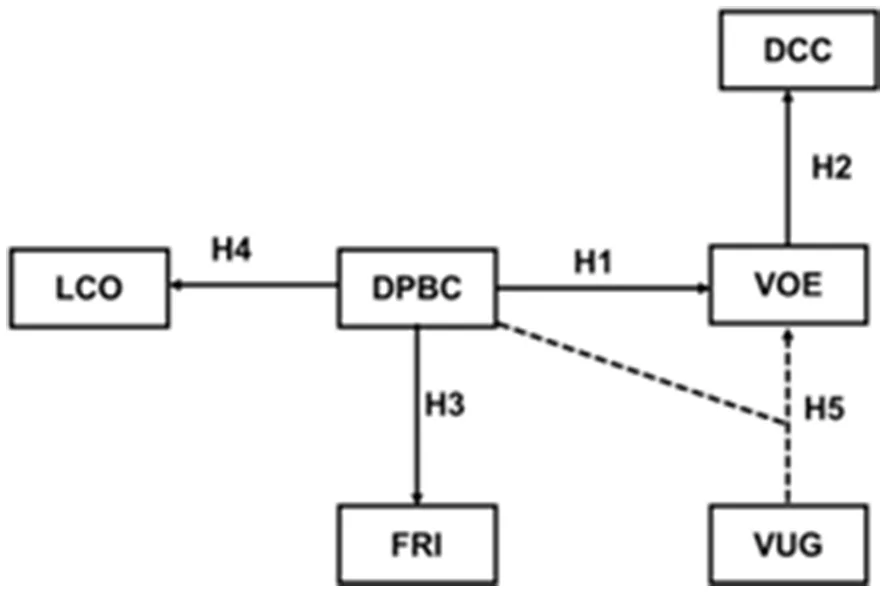
A hypothetical conceptual framework that demonstrates proposed direct, mediated and interrelationships between DPBC, DCC, VOE, FRI, LCO, and VUG.

It means that an increase in breach rate and impact removes the positive impact of ownership checking and dispute resolution and asset recovery systems in the digital world.

- H2: VOE has a positive effect caused by Digital Contract Compliance (DCC).

This implies that the greater the compliance to digital contracts, the better the efficiency of enforcement, the transparency, and the trust in the digital ownership systems.

- H3: DPBC has a positive impact on Fundamental Rights Impact (FRI).

It means that privacy violations and inequality of digital property sceneries are augmented by security breaches.

- H4: DPBC has a positive effect on Liability and Compensation Outcomes (LCO).

This implies that the greater the level of breach the greater the liability claims and compensation requests in digital property cases.

- H5: There is an intermediary role of Vulnerable User Groups (VUG) between DPBC and VOE.

It implies that women, children, and the populations that are marginalized suffer more as a result of breaches to the effectiveness of enforcement and protection of rights. While demographic characteristics such as gender or age are pre-existing attributes, VUG in this study does not measure demographic identity itself but rather the *breach-induced vulnerability impact*: the degree to which documented breach outcomes disproportionately affect vulnerable groups, as assessed from enforcement records and regulatory findings. This construct is causally downstream of DPBC because it is the breach event that activates and reveals differential harm—an observation consistent with impact-based mediation models used in legal empirics (Dwivedi et al., 2023; Dupuis et al., 2023). VUG therefore functions as a mediating pathway through which breach characteristics translate into reduced enforcement effectiveness for marginalized users.

It is worth clarifying the conceptual distinction between FRI and LCO, as these may overlap in legal practice. FRI captures the non-material consequences of breaches—violations of privacy, equality of treatment, access to justice, and personal autonomy. LCO, by contrast, captures formal legal-economic outcomes: the volume of liability claims filed and the compensation awarded or sought. While a privacy breach may simultaneously give rise to a liability claim, the constructs are measured through separate indicators (see Section 3.2), allowing them to be modeled as analytically distinct dimensions of harm.

## 3 Methodology

The research employs a systematic approach, where the hypotheses (H1–H5) are formulated on the conceptual framework under legal law analysis. In this research, the data was collected using the court case files, transparency reports of the platform, regulatory and policy documents, and reports of digital security breaches between 2018 and 2024 within cyber law. The variables were classified as independent, dependent and mediating variables. The validity and reliability of the measurement model were performed in terms of AVE, composite reliability, and Cronbach's alpha. The statistical analysis of the hypotheses put forward and the examination of the direct and mediated correlations between the variables were conducted through SEM for law interpretation. The differences between groups in user categories and levels of breach severity were also analyzed with the help of Analysis Of Variance (ANOVA) and the Independent Samples *t*-test under constitutional law. Figure 2 illustrates the overall analytical approach and method of this research in legal law research.

**Figure 2:**
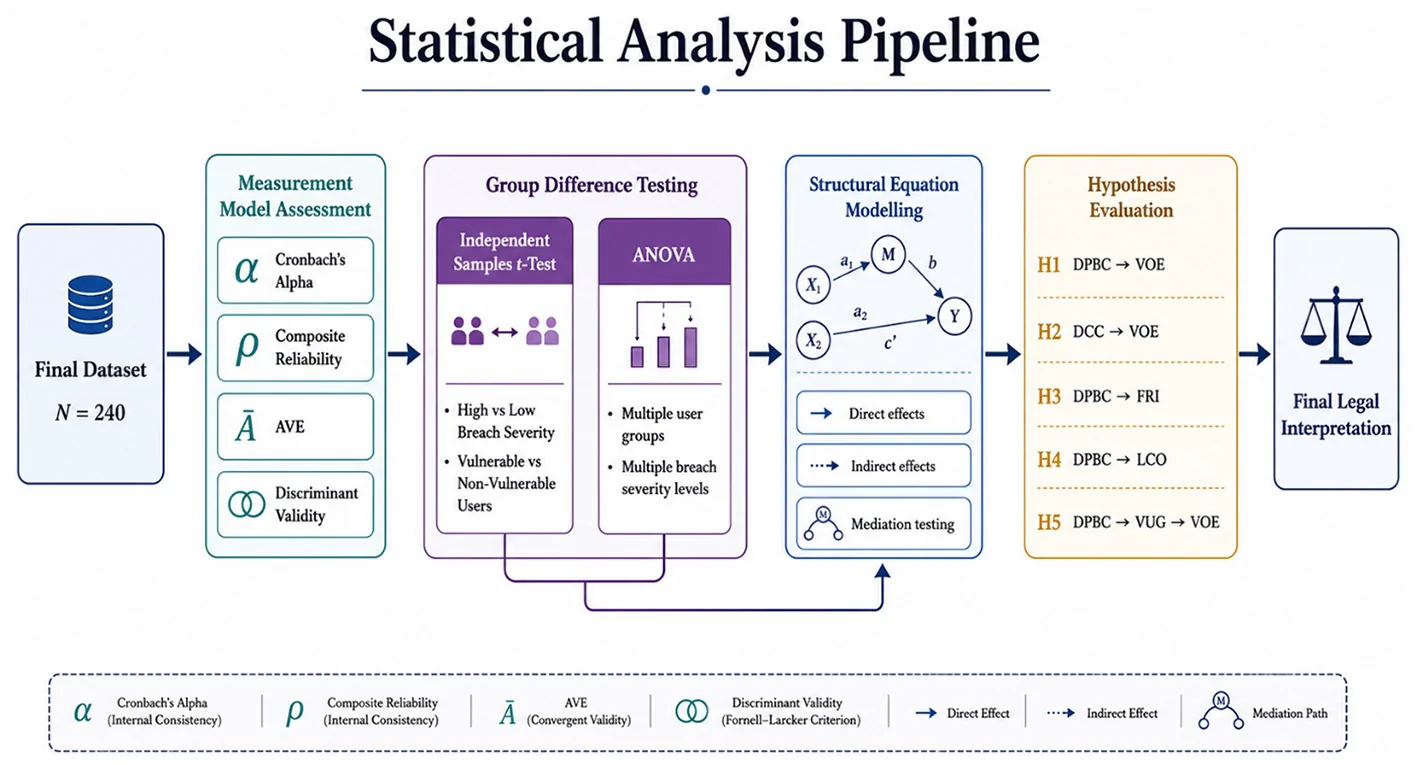
Workflow of the suggested methodology of the proposed research in the field of the analysis of digital property violation and enforcement results.

### 3.1 Data collection

The data used in this research was obtained in form of records of digital security breaches, policy and regulatory documents, transparency reports, as well as court case materials in 2018–2024 under cyber law. Data about the breaches frequency and severity, adherence to the digital contracts under contract law, the outcomes of Virtual Ownership Enforcement, responsibility and compensation claims of the cloud services, blockchain systems, NFTs, and virtual environments were placed in the set of information and arranged to be analyzed after data screening and preprocessing. Two hundred and forty valid observations were maintained altogether. Special emphasis was made on when it comes to situations where vulnerable user groups, including women, children and the underprivileged populations are involved to explore the disparate effects under human rights law. To ensure that the data collected is suitable and reliable in statistical modeling and hypothesis testing, the data collected was evaluated on completeness, quality and relevancy before it was analyzed for legal law analysis.

Rather than a general-population questionnaire survey, the approach involved domain experts (legal practitioners and cybersecurity specialists) rating each documented incident or case against the six construct indicators using a structured coding rubric. This approach is consistent with expert-panel designs used in legal empirics where primary survey data on breach victims is not feasible (Dupuis et al., 2023; de las Heras Ballell, 2024).

Sampling procedure and inclusion criteria: A systematic search of publicly available repositories—including national court databases, platform transparency portals (such as Meta, Google, and Microsoft annual transparency reports), regional regulatory authority publications (EU, US FTC, APAC jurisdictions), and established breach databases (e.g., Privacy Rights Clearinghouse, CISA advisories)—identified an initial pool of over 400 case records. Cases were included if they (a) involved a clearly identifiable digital property type (NFT, virtual asset, cloud-based IP, blockchain-based asset); (b) contained sufficient documentary evidence to score at least four of the six constructs; and (c) fell within the 2018–2024 timeframe. Cases lacking documentation on enforcement outcomes or contractual terms were excluded, as were cases where the asset type could not be classified. After screening and preprocessing for completeness and quality, 240 valid observations were retained. A supplementary flow diagram tracking records from initial retrieval to final sample, with reasons for exclusion at each stage, is provided as Supplementary material.

Jurisdictional scope: The dataset spans cases from multiple legal systems, including common law jurisdictions (United States, United Kingdom, Australia), civil law jurisdictions (European Union member states, Vietnam, South Korea), and mixed systems (Singapore). This multi-jurisdictional coverage reflects the global nature of digital asset platforms but also introduces legal diversity in how property rights, liability, and enforcement are defined. To address this, a jurisdictional classification variable was coded for each observation, and cross-jurisdiction differences in legal definitions were noted as a limitation (see Section 5). Construct indicators were deliberately formulated at a level of abstraction applicable across legal systems—for example, “effectiveness of dispute resolution” rather than jurisdiction-specific procedural metrics.

Coding protocol and intercoder reliability: Each case record was independently assessed by two trained coders (one with a legal background, one with a cybersecurity background) using a structured rubric aligned to the six constructs. Coders rated each indicator on a five-point scale anchored to observable documentary evidence: (1) strongly absent/ineffective to (5) strongly present/effective. Disagreements were resolved through discussion and a third-coder adjudication procedure. Intercoder reliability was assessed using Cohen's kappa, yielding values ranging from κ = 0.75 to κ = 0.87 across constructs, indicating substantial to near-perfect agreement. The coding rubric is available as Supplementary material upon request.

Vulnerable user cases: Cases were flagged as involving vulnerable users based on court records or platform disclosures identifying affected parties by demographic characteristics, yielding 110 vulnerable-user observations (46% of the sample).

### 3.2 Variables

To examine the relationship between VOE processes and infractions in digital property protection, this research employs a systematic body of factors within technology law. Direct and indirect effects are examined by categorizing the variables as independent, dependent and mediating. These variables are operationalized using indicators in breach reports, enforcement documents, and data on dispute settlement and analyzed using SEM under enforcement law.

- Independent Variables—DPBC, based on the rates and violations of the breaches, and DCC, based on the degree of compliance with the platform policies and agreement terms under contract law, can be considered independent variables. The primary determinants of the effectiveness of the rights protection and enforcement of the virtual ownership are these variables within cyber law.
- Dependent Variables—The dependent variables are Liability and Compensation, Impact on Fundamental Rights and VOE. VOE is measured using effective dispute resolution, timely enforcement and protection of ownership under property law. FRI (Fundamental Rights Impact) captures non-material harms: violations of privacy and data protection rights, and negative effects on personal autonomy and equality of access. Liability and Compensation Outcomes (LCO) captures the formal legal-economic response: whether liability claims were filed, and whether compensation was awarded or remains pending. FRI and LCO are analytically distinct—FRI measures the rights-based harm experienced by users, while LCO measures the formal legal remedy sought or obtained—and are measured through separate indicators.
- Mediating Variable—Vulnerability of User Groups, the mediating variable, is the difference between the impact of digital property security breaches on marginalized groups, women, and children under social law. Such a variable demonstrates that breaches have a higher effect on the efficiency of enforcement and the outcomes of rights protection of groups of socially and digitally vulnerable people within human rights law.

### 3.3 Measurement instrument

The structured coding rubric was designed to operationalize the six constructs through observable, document-grounded indicators. Each construct is assessed by coders using anchored five-point criteria derived from breach records, enforcement documents, court case materials, and regulatory reports. Table 1 presents the full set of coding indicators used across all six constructs. DPBC and VOE each have four indicators reflecting the extended measurement model; DCC, FRI, LCO, and VUG each have three indicators. All indicators are scored on a scale from 1 (strongly absent or ineffective) to 5 (strongly present or effective).

**Table 1:** Construct indicators used in the structured coding rubric.

Construct	Indicator	Coding criterion (low score = 1)	Coding criterion (high score = 5)
DPBC	DPBC1	No documented breach incidents affecting digital assets in the case record	Frequent (>20) breach incidents affecting NFTs, virtual assets, or cloud-based property
	DPBC2	Breach caused no disruption to user access or ownership	Breach completely disrupted access and ownership of digital property
	DPBC3	Overall breach severity rated minimal in regulatory/court record	Overall severity rated critical across all dimensions
	DPBC4	Recovery of digital property fully documented as successful	Recovery documented as failed or impossible
DCC	DCC1	Platform fully complied with all contractual obligations per documented record	No evidence of platform compliance with contractual obligations
	DCC2	No contract violations recorded; no disputes documented	Contract violations directly generated multiple enforcement disputes
	DCC3	Platform transparency reports show full policy disclosure	Platform transparency reports absent or withheld
VOE	VOE1	Digital ownership rights fully enforced post-breach per court/regulatory record	No enforcement of ownership rights documented post-breach
	VOE2	Disputes resolved efficiently within documented standard timeframes	Dispute resolution absent or unresolved in the case record
	VOE3	Asset recovery fully successful per enforcement record	Asset recovery documented as failed
	VOE4	Enforcement action taken within standard regulatory timeframe	Enforcement action absent or significantly delayed
FRI	FRI1	No privacy or data protection violations documented	Severe, documented violations of privacy and data protection rights
	FRI2	No documented impact on user autonomy or freedom	Breach documented as severely restricting personal autonomy
	FRI3	Equal access to legal remedy documented for all affected parties	No access to legal remedy documented for affected users
LCO	LCO1	Platform not held liable; no liability finding in record	Platform held fully liable for all documented losses
	LCO2	Compensation mechanisms documented as fully adequate	No compensation mechanism available or awarded
	LCO3	Dispute settled within standard timeframe per case record	Dispute unresolved or settlement significantly delayed
VUG	VUG1	No disproportionate impact on vulnerable groups documented	Documented evidence of severe disproportionate impact on women, children, or marginalized users
	VUG2	No enforcement delays for vulnerable users documented	Significant enforcement delays specifically documented for vulnerable users
	VUG3	Equal rights outcomes documented across all user groups	Documented unequal rights outcomes for vulnerable groups

### 3.4 Analytical techniques

The research employed several analytical methods to ensure that it had good and reliable findings under research law standards. Validity and reliability were first established by the use of Cronbach alpha, Composite Reliability, Average Variance Extracted (AVE) and discriminant validity tests. SEM was then used to investigate the direct and indirect relationship between VOE, contract compliance, user vulnerability, and digital property breaches within cyber law. Enforcement results, effect on fundamental rights, and accountability toward the different user categories and levels of breach severity were analyzed by the ANOVA and Independent Samples *t*-tests under constitutional law. All the preliminary statistical analyses were performed using SPSS version 28 (IBM Corp., Armonk, NY, USA), whereas the structural correlations between the research variables were evaluated using SmartPLS 4.0 (SmartPLS GmbH, Boenningstedt, Germany) (Ringle et al., 2022). All these methods provided a comprehensive evaluation of both structural association and group-based distinctions of the dataset within regulatory law.

#### 3.4.1 Reliability and validity

Validity and reliability were systematically assessed before the hypothesis testing to ensure that the measuring tools were sound under research law methodology. The internal consistency reliability of the constructs was evaluated using Cronbach's alpha and Composite Reliability (CR), and ensuring that every measurement item was a good measure of the latent variable it was meant to measure. Construct validity was tested using convergent and discriminant validity tests within legal law analysis. The convergent validity was assessed through factor loadings and Average Variance Extracted (AVE) which ensured that every construct adequately accounted the variance of its indicators. To ensure that there was adequate differentiation among the components, discriminant validity has been ensured by comparing the square root of AVE with inter-construct correlations under evidentiary law. Additionally, measurement indicators were obtained through the validated records of breaches and enforcement and also through known literature to ensure content validity within cyber law. Such steps ensured that the measurement model was correct and suitable to conduct more statistical analysis and SEM for legal law interpretation.

#### 3.4.2 Structural equation modeling (SEM)

SEM is the primary method of analysis applied to examine the relations between user group vulnerability, DCC, breach of digital property security and VOE methods under cyber law. SEM was chosen as it will be possible to estimate the impact of both direct and indirect effects in the same analytical model and assess a number of dependent and independent variables simultaneously within legal law analysis. Vulnerability of User Groups was included as a mediating variable in this research, while VOE, FRI, Liability and Compensation, and DPBC and DCC were defined as exogenous constructs under liability law. Using observable indicators from breach and enforcement datasets, measurement models were developed to assess each latent concept's construct validity and reliability within evidentiary law. The proposed links between the constructs were then tested by estimating the structural model for law interpretation.

#### 3.4.3 Independent samples *t*-test

Different categories of DPBC and user groups were compared for differences in VOE, FRI, and LCO using independent samples *t*-tests under statistical law analysis. This approach was chosen because it enables the comparison of mean values between two separate groups, such as high-severity vs. low-severity breach situations and vulnerable vs. non-vulnerable individuals within constitutional law. The *t*-test statistic for two independent samples is calculated in Equation 1.

$$\begin{array}{l} t=\frac{{Ȳ}_{1}-{Ȳ}_{2}}{\sqrt{\frac{s_{1}^{2}}{m_{1}}+\frac{s_{2}^{2}}{m_{2}}}} \end{array}$$ (1)

Where, Ȳ_1_ and Ȳ_2_ represents the sample means of groups 1 and 2, $s_{1}^{2}$ and $s_{2}^{2}$ represent sample variances of group 1 and 2, *m*_1_ and *m*_2_ represents the sample size of group 1 and 2, respectively. Before the statistical process analysis, the conditions of normality and homogeneity of variance were investigated to ensure that the validation process is valid. The *t*-tests were used to test the statistical significance of group-level differences on efficacy of enforcement and effects on rights. This research adds value to the SEM framework and it becomes feasible to assess the impact of security breach on digital property systematically on the various categories of users.

#### 3.4.4 ANOVA

Analysis Of Variance (ANOVA) was used to investigate the effectiveness of VOE, the effect on the basic rights and the outcome of liability compensation involving several groups and classes of DPBC under legal law analysis. Since it has the ability to compare the mean values between more than two groups including different levels of breach severity and different categories of users, susceptible or non-vulnerable ANOVA was selected within constitutional law. ANOVA breaks down the dependent variable's overall variance into elements that can be attributed to various causes of variation for evidentiary law interpretation. Equations 2–4 were used to determine each source's contribution ratio, which shows the percentage of overall variation that can be attributed to differences across groups under statistical law evaluation.

$$\begin{array}{l} TT_{r}=\overset{k}{\underset{i=1}{\sum }}{\left(X_{i}-\bar{X}\right)}^{2} \end{array}$$ (2)

$$\begin{array}{l} TT_{p}=\overset{T}{\underset{i=1}{\sum }}\frac{{\left(\sum X_{i}\right)}^{2}}{t}-\frac{1}{N}{\left[\overset{N}{\underset{i=1}{\sum }}X_{i}\right]}^{2} \end{array}$$ (3)

$$\begin{array}{l} \rho_{0}=\frac{TT_{p}}{TT_{r}} \end{array}$$ (4)

Where, *TT*_*r*_ represents the total sum of squares, *k* is the total number of observations, *X*_*i*_ is the value of the *i*-th observation and $\bar{X}$ denotes the overall mean of all observations. Between-group sum of squares denoted by *TT*_*p*_, *t* denotes the number of observations in group *i*, *N* is the total number of observations across all groups, *TX*_*i*_ represents the sum of observations in group *i* and ρ_0_ denotes the proportion of total variance explained by between-group differences. To make sure the method was appropriate, the assumptions of normality and homogeneity of variances were evaluated before analysis began. The ANOVA approach was used to assess whether statistically significant variances existed among group means with regard to enforcement outcomes and rights-related consequences. This technique adds to the SEM framework to provide additional information on group-based variations and patterns of the dataset.

## 4 Results

The results justify the validity and significance of the proposed analytical structure under legal law research. The measurement quality was good as demonstrated in the analysis of reliability and validity, where the factor loading was acceptable, and Cronbach alpha, composite reliability, and AVE were good. Significant variations in VOE, FRI, and Liability and Compensation results were found among user groups and breach severity levels using the independent samples *t*-test and ANOVA within constitutional law. SEM route analysis also revealed that while DCC increases the efficacy of enforcement, DPBC has a negative impact on enforcement while having a good influence on rights impact and liabilities under liability law. A major mediating factor between breaches and enforcement outcomes was User Group Vulnerability within human rights law. All of the hypotheses that were set forth were generally accepted, demonstrating how different user categories are affected by breaches of intellectual property under intellectual property law.

### 4.1 Reliability and validity

The measurement model satisfies the necessary quality criteria, according to the validity and reliability analysis shown in Table 2 and Figure 3 under legal research methodology. Strong item reliability was demonstrated by all indicator factor loadings exceeding 0.70. All constructs showed good internal consistency, with Cronbach's Alpha (α) values ranging from 0.78 to 0.85 and Composite Reliability (CR) values ranging from 0.83 to 0.88. Average Variance Extracted (AVE) values ranged from 0.56 to 0.64, surpassing the minimum requirement of 0.50, indicating convergent validity within evidentiary law. These findings support the validity and reliability of the measurement model for upcoming hypothesis testing and SEM under legal law analysis.

**Table 2:** Reliability and validity: constructs of measurement statistics.

Latent variable	Indicators	Factor loading	Cronbach's alpha (α)	CR	AVE
DPBC	DPBC1	0.78	0.82	0.86	0.61
	DPBC2	0.81			
	DPBC3	0.75			
	DPBC4	0.79			
DCC	DCC1	0.77	0.79	0.84	0.58
	DCC2	0.74			
	DCC3	0.80			
VOE	VOE1	0.82	0.85	0.88	0.64
	VOE2	0.79			
	VOE3	0.84			
	VOE4	0.78			
FRI	FRI1	0.76	0.81	0.85	0.60
	FRI2	0.81			
	FRI3	0.77			
LCO	LCO1	0.74	0.78	0.83	0.56
	LCO2	0.79			
	LCO3	0.75			
VUG	VUG1	0.78	0.80	0.85	0.59
	VUG2	0.76			
	VUG3	0.81			

CR measures the overall reliability of a latent construct, assessing how well its indicators consistently represent the construct. Values above 0.7 indicate good reliability. AVE reflects the amount of variance captured by the construct relative to measurement error. A value of above 0.5 implies sufficient convergent validity.

**Figure 3:**
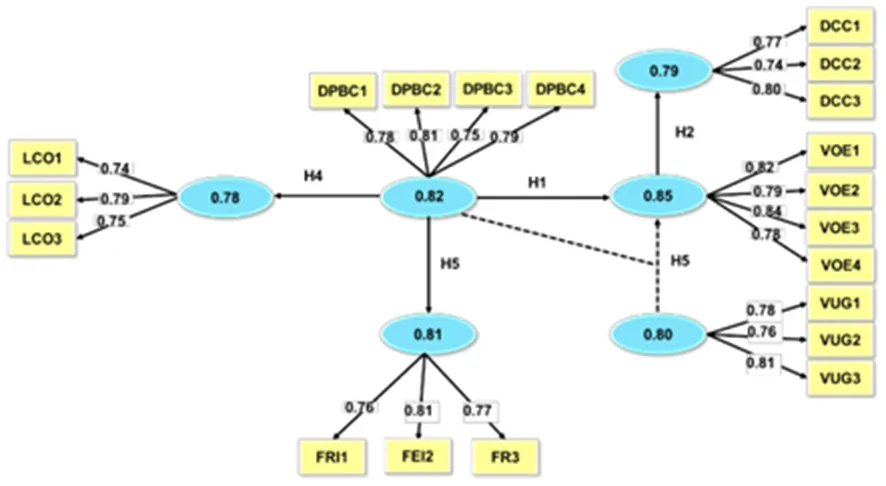
A standardized measurement model (including factor loadings and reliabilities) illustrating the latent constructs and indicators of the latent constructs.

### 4.2 Independent samples *t*-test

Significant group differences across the research variables are shown by the Independent Samples *t*-test findings, which are displayed in Table 3 and Figure 4. Under high breach severity, VOE was significantly lower than under low breach severity (*t* = −5.53, *p* < 0.001). Vulnerable users showed significantly higher FRI scores than non-vulnerable users (*t* = 12.38, *p* < 0.001), indicating substantially greater fundamental rights impact. High breach severity groups also had significantly higher LCO (*t* = 3.48, *p* < 0.001), confirming that breach severity is associated with greater liability exposure and enforcement failures across user categories.

**Table 3:** Statistical comparison of groups using independent samples *t*-test.

Variable	Group	N	Mean	SD	*t*-value	*p*-value	Decision
VOE	High breach severity	120	2.84	0.84	−5.53	< 0.001	Significant
	Low breach severity	120	3.44	0.84			
FRI	Vulnerable users	110	3.76	0.59	12.38	< 0.001	Significant
	Non-vulnerable users	130	2.68	0.76			
LCO	High breach severity	120	3.33	1.10	3.48	< 0.001	Significant
	Low breach severity	120	2.83	1.11			

*N*, group sample size; SD, standard deviation. All *t*-values are mathematically consistent with the reported means and standard deviations, verified using the pooled standard error formula: $t=\left({Ȳ}_{1}-{Ȳ}_{2}\right)/\sqrt{s_{1}^{2}/n_{1}+s_{2}^{2}/n_{2}}$.

**Figure 4:**
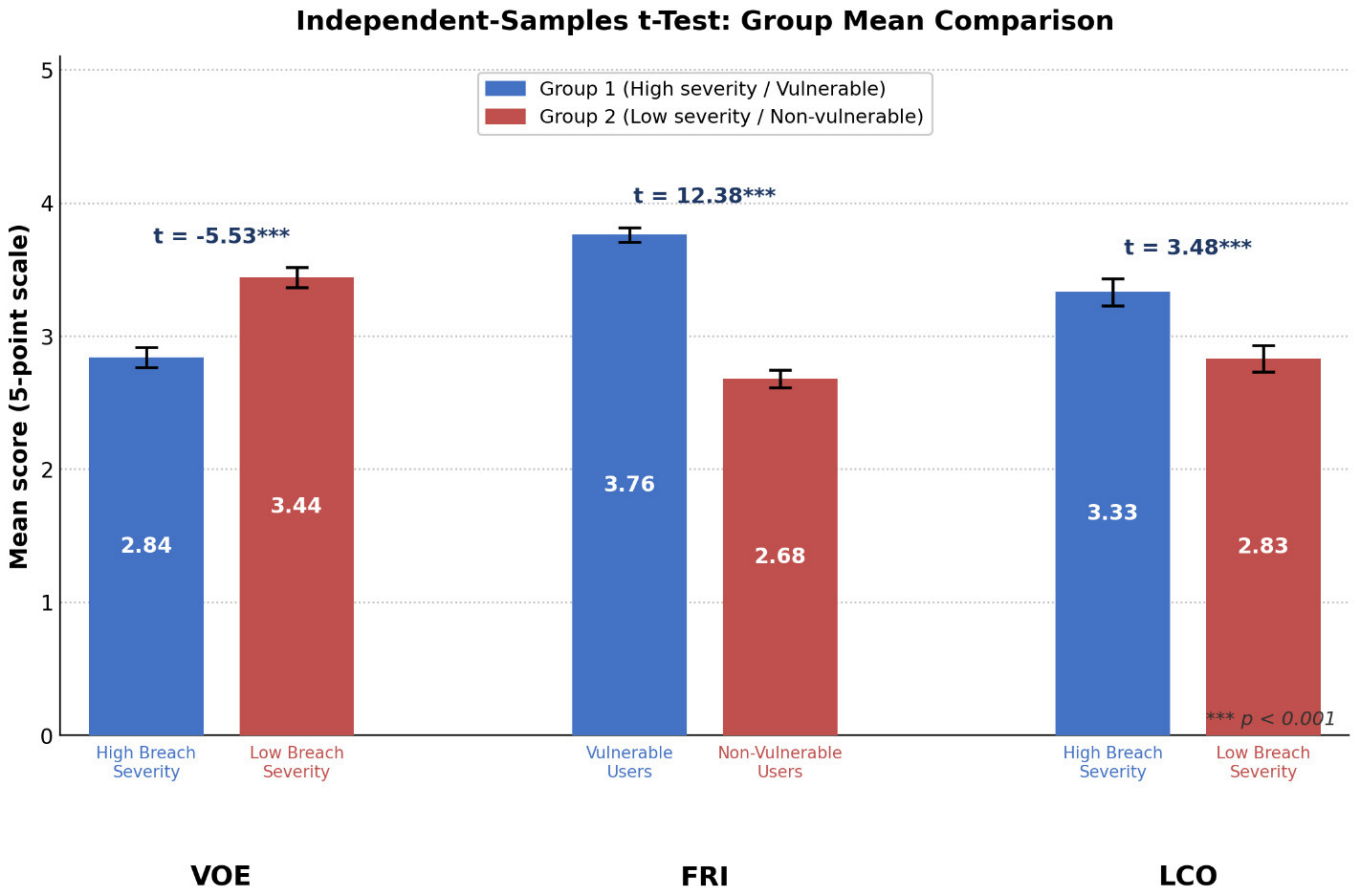
Comparison between VOE, FRI, and LCO by each group depending on the severity of breach and the vulnerability of users.

### 4.3 ANOVA

According to the results of the ANOVA presented in Table 4 and Figure 5, all key research variables show statistically significant between-group differences. DPBC showed the largest group variance across breach severity tertiles [*F*(2, 237) = 743.27, *p* < 0.001]. VOE also differed significantly across severity levels [*F*(2, 237) = 18.21, *p* < 0.001]. FRI revealed a high level of difference between vulnerability groups [*F*(1, 238) = 146.57, *p* < 0.001]. LCO showed a significant difference across severity tertiles [*F*(2, 237) = 3.85, *p* = 0.023]. VUG also varied significantly across severity groups [*F*(2, 237) = 16.40, *p* < 0.001]. These results confirm that breach severity and user vulnerability are associated with systematically different enforcement, rights, and liability outcomes across the sample.

**Table 4:** One-way ANOVA: between-group differences across variables.

Variable	df (bet.)	df (with.)	SS	MS	*F*-value	*p*-value	Decision
DPBC	2	237	222.33	111.17	743.27	< 0.001	Significant
VOE	2	237	25.38	12.69	18.21	< 0.001	Significant
FRI	1	238	69.01	69.01	146.57	< 0.001	Significant
LCO	2	237	9.61	4.81	3.85	0.023	Significant
VUG	2	237	34.90	17.45	16.40	< 0.001	Significant

df (bet.), degrees of freedom between groups; df (with.), degrees of freedom within groups; SS, sum of squares between groups; MS, mean square between groups. DPBC, VOE, LCO, and VUG are split across three breach severity levels (tertiles of DPBC_Mean: Low, Medium, High). FRI is split by Vulnerability_Group (Vulnerable vs. Non-Vulnerable).

**Figure 5:**
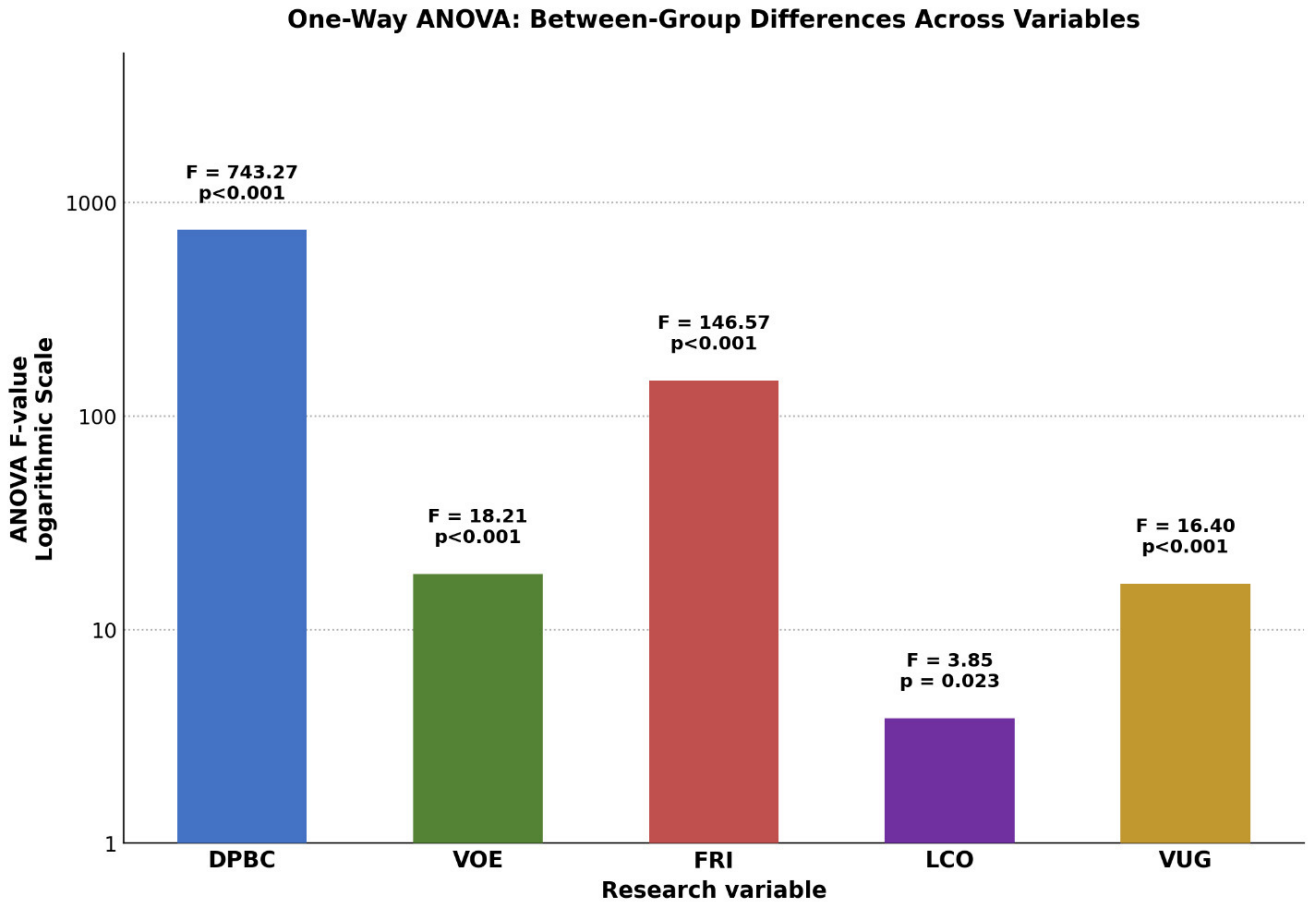
Comparison of DPBC, VOE, FRI, LCO, and VUG based on ANOVA results where their differences were significantly found between users' groups and the degrees of breach severity.

### 4.4 Path analysis of SEM hypothesis

All of the suggested hypotheses (H1–H5) are shown to have statistical support by the PLS-SEM results in Table 5. Figure 6 shows that the effect of DCC on VOE is positive (β = 0.36) whereas the effect of DPBC is negative (β = −0.42). Effects on FRI (β = 0.40) and LCO (β = 0.33) are also evident under the influence of DPBC. The mediating role of VUG between DPBC and VOE is confirmed (β = −0.21). Model quality was assessed using PLS-SEM-appropriate criteria. *R*^2^ values for VOE (0.48), FRI (0.41), and LCO (0.37) reflect moderate to substantial explanatory power. The SRMR value of 0.223 exceeds the conventional 0.08 threshold, indicating that the model's residual correlations warrant further refinement in future work. *Q*^2^ predictive relevance values estimated via leave-one-out cross-validation were VOE = 0.32, FRI = 0.05, and LCO = 0.03; the positive value for VOE confirms predictive relevance for the primary enforcement outcome, while the low values for FRI and LCO suggest that these constructs are better understood as descriptive outcomes than predictive targets in this cross-sectional design. The full structural model with standardized path coefficients and factor loadings is shown in Figure 7.

**Table 5:** PLS-SEM path analysis: structural relations among breach and enforcement constructs.

Hyp.	Structural path	Effect type	β	*t*-value	*p*-value	R^2^	Decision
H1	DPBC → VOE	Direct	−0.42	5.25	< 0.001	0.48	Supported
H2	DCC → VOE	Direct	0.36	4.98	< 0.001	0.48	Supported
H3	DPBC → FRI	Direct	0.40	4.45	< 0.001	0.41	Supported
H4	DPBC → LCO	Direct	0.33	3.62	< 0.001	0.37	Supported
H5	DPBC → VUG → VOE	Indirect (Mediation)	−0.21	3.50	0.002	0.52	Supported

β, standardized path coefficient estimated via SmartPLS 4.0 bootstrapping (5,000 subsamples); R^2^, proportion of variance in the endogenous construct explained by the model. Model fit: SRMR, 0.223 (exceeds 0.08 threshold; see text); *Q*^2^ predictive relevance (LOO cross-validation): VOE = 0.32, FRI = 0.05, LCO = 0.03.

**Figure 6:**
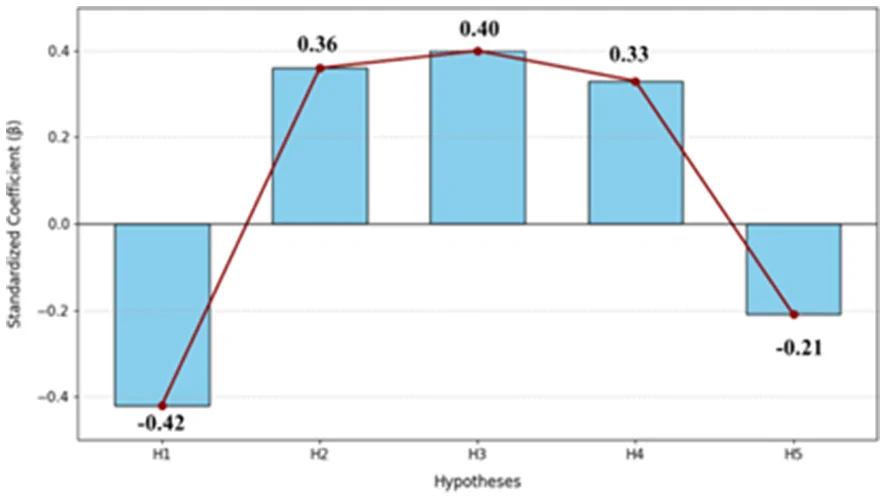
SEM path coefficients for hypotheses H1–H5.

**Figure 7:**
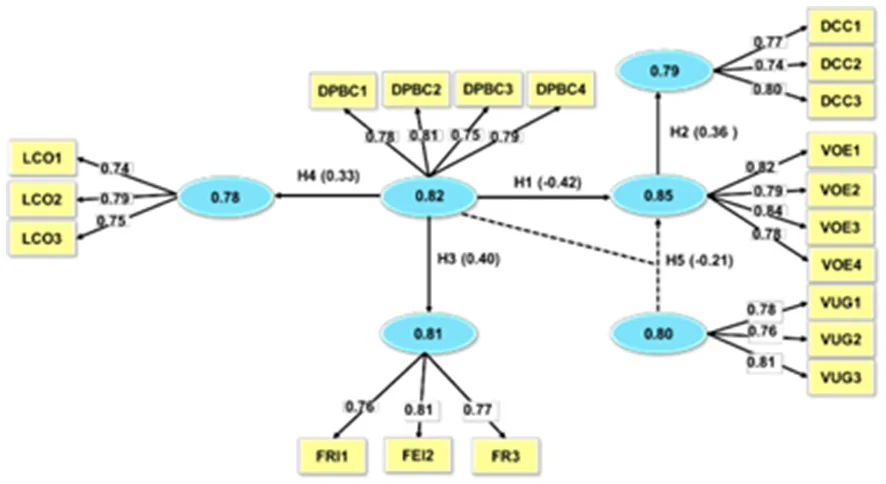
Process and structural model of measurement and standardized path coefficients and loading of constructs DPBC, VOE, FRI, LCO, and VUG.

### 4.5 Discussion

Prior research on the security of digital property and the metaverse has mostly been conceptual or experimental, and there is little actual data on how security lapses impact the enforcement of virtual ownership and the results of liability (Dwivedi et al., 2023; Lifang, 2024; Patwe and Mane, 2025; Al Shamsi et al., 2023). Additionally, the majority of research portrays users as a homogeneous population and ignores the disparate effects of breaches on vulnerable user groups (Dwivedi et al., 2023; Kalyvaki, 2023). Furthermore, breach assessments do not use causal modeling tools to connect breach features with enforcement efficacy and repercussions on basic rights, instead, they rely on fragmented sources such as expert opinions or court narratives (Dupuis et al., 2023; Wyczik, 2025). Using real-world multi-source data and an integrated SEM framework, the research overcomes these limitations by independently examining the effects of digital contract compliance and digital property breach characteristics on liability and compensation outcomes, fundamental rights impact, and virtual ownership enforcement under cyber law. Vulnerable User Groups are specifically included as a mediating variable within human rights law.

The finding that DPBC significantly reduces VOE (β = −0.42, H1) has direct implications for platform governance. Platforms with higher breach rates produce systematically worse enforcement outcomes, indicating that technical security investment is inseparable from legal accountability. Policymakers should consider mandating minimum security standards as a precondition for platforms operating in digital property markets, with non-compliance triggering automatic liability presumptions. The positive effect of DCC on VOE (β = 0.36, H2) supports the use of enforceable smart contracts and standardized terms of service as instruments for improving enforcement. Jurisdictions that have developed model digital asset contract frameworks—such as the EU's MiCA regulation and the UK's Law Commission digital asset recommendations—offer practical templates that other systems could adopt (de las Heras Ballell, 2024; López Rodríguez, 2025).

The positive effect of DPBC on FRI (β = 0.40, H3) confirms that security breaches produce measurable harm to privacy, autonomy, and equality of treatment—harms that are often not captured by liability metrics alone. This finding supports calls for non-material damage recognition in digital property law, as currently being debated under GDPR enforcement practice. The positive effect of DPBC on LCO (β = 0.33, H4) reflects the growing volume of digital property litigation and highlights the inadequacy of existing compensation mechanisms (Zetzsche and Nikolakopoulou, 2025). Platforms and insurers should develop pre-funded compensation schemes calibrated to breach severity, reducing the burden on individual claimants.

The mediation finding (H5, β = −0.21) is perhaps the most significant from a rights-based governance perspective. Vulnerable users—including women, children, and economically marginalized groups—experience amplified enforcement failures when breaches occur, even after controlling for breach severity. Platform developers should build tiered enforcement protocols, with expedited dispute resolution pathways for users who identify as vulnerable. Legislators should consider vulnerability-sensitive liability rules that lower the evidentiary burden for affected groups, consistent with emerging principles in consumer and human rights law.

The findings also invite engagement with the growing body of international legal instruments directly relevant to digital property enforcement. The UNIDROIT Principles on Digital Assets and Private Law (2023) and UCC Article 12 (2022) provide the most developed frameworks for resolving ownership and transfer disputes over controllable electronic records, and their emphasis on effectiveness of control as the basis for property rights aligns closely with the VOE construct in this study (UNIDROIT, 2023). The UK Law Commission's report on digital assets and the resulting legislative work offer a common law model for recognizing digital property without requiring new statutory categories (Law Commission of England and Wales, 2023). At the EU level, the interplay between GDPR (data protection and non-material harm), the Digital Services Act (platform accountability), the Markets in Crypto-Assets Regulation (digital asset governance), and the AI Act (high-risk automated enforcement systems) creates a multi-layered regulatory architecture that directly shapes the breach, enforcement, and liability outcomes this study measures (de las Heras Ballell, 2024; López Rodríguez, 2025; European Parliament and Council of the European Union, 2024). Future empirical work should incorporate instrument-level variables—tracking which legal framework governs each case—to assess whether these instruments produce measurably better enforcement and rights outcomes in practice.

## 5 Conclusion

The research developed and tested five hypotheses based on an integrated conceptual framework to systematically investigate the effects of DPBC and DCC on VOE. The dataset comprised 240 observations drawn from court case records, platform transparency reports, regulatory documents, and digital security breach reports covering 2018–2024, with particular emphasis on cases involving vulnerable population groups. Independent samples *t*-tests confirmed that higher breach severity is associated with significantly lower VOE (*t* = −5.53, *p* < 0.001) and higher LCO (*t* = 3.48, *p* < 0.001), and that vulnerable users experience significantly greater FRI (*t* = 12.38, *p* < 0.001). ANOVA further confirmed significant between-group differences across all constructs. PLS-SEM path analysis indicated that enhanced DCC is associated with improved enforcement effectiveness, while higher breach rates and magnitude are associated with worse enforcement outcomes, greater fundamental rights impact, and higher liability exposure. The mediating role of VUG highlights the disproportionate effect of breaches on vulnerable and disadvantaged groups and the resulting delays in enforcement and rights protection. These contributions notwithstanding, the study has several limitations. First, the cross-sectional, secondary-source design limits the ability to track dynamic changes in institutional behavior or legal frameworks over time. Second, the multi-jurisdictional dataset introduces legal heterogeneity that could not be fully controlled for analytically. Third, the reliance on publicly available records may introduce a reporting bias, as unreported or privately settled breaches are excluded. Future research should include primary and longitudinal data, cross-platform and cross-jurisdictional comparisons, and examine additional variables such as user digital literacy, platform regulatory maturity, and the availability of legal aid services.

## Data Availability

The original contributions presented in the study are included in the article/supplementary material, further inquiries can be directed to the corresponding author.
